# Regulatory Effect of Irresistin-16 on Competitive Dual-Species Biofilms Composed of *Streptococcus mutans* and *Streptococcus sanguinis*

**DOI:** 10.3390/pathogens11010070

**Published:** 2022-01-06

**Authors:** Xiangyu Hu, Min Wang, Yan Shen, Lingjun Zhang, Yihuai Pan, Yan Sun, Keke Zhang

**Affiliations:** Institute of Stomatology, School of Stomatology, Wenzhou Medical University, Wenzhou 325035, China; xiangyuhu@wmu.edu.cn (X.H.); minwang@wmu.edu.cn (M.W.); sissi960810@gmail.com (Y.S.); zlj2312@163.com (L.Z.)

**Keywords:** irresistin-16, dual-species biofilms, *Streptococcus mutans*, *Streptococcus sanguinis*, regulatory effect, cariogenic virulence

## Abstract

Based on the ecological plaque hypothesis, suppressing opportunistic pathogens within biofilms, rather than killing microbes indiscriminately, could be a biofilm control strategy for managing dental caries. The present study aimed to evaluate the effects of irresistin-16 (IRS-16) on competitive dual-species biofilms, which consisted of the conditional cariogenic agent *Streptococcus mutans* (*S. mutans*) and oral commensal bacteria *Streptococcus sanguinis* (*S. sanguinis*). Bacterial growth and biofilm formation were monitored using growth curve and crystal violet staining, respectively. The microbial proportion was determined using fluorescence in situ hybridization. A 2, 5-diphenyltetrazolium bromide assay was used to measure the metabolic activity of biofilms. Bacterial/extracellular polysaccharide (EPS) dyeing, together with water-insoluble EPS measurements, were used to estimate EPS synthesis. A lactic acid assay was performed to detect lactic acid generation in biofilms. The cytotoxicity of IRS-16 was evaluated in mouse fibroblast L929 cells using a live/dead cell viability assay and cell counting kit-8 assay. Our results showed that IRS-16 exhibited selective anti-biofilm activity, leading to a remarkable survival disadvantage of *S. mutans* within competitive dual-species biofilms. In addition, the metabolic activity, EPS synthesis, and acid generation of dual-species biofilms were significantly reduced by IRS-16. Moreover, IRS-16 showed minimal cytotoxicity against mouse fibroblast L929 cells. In conclusion, IRS-16 exhibited remarkable regulatory effects on dual-species biofilms composed of *S. mutans* and *S. sanguinis* with low cytotoxicity, suggesting that it may have potential for use in caries management through ecological biofilm control.

## 1. Introduction

Dental caries is a significant public health problem worldwide, and it imposes a heavy economic burden on individuals and society [1]. In 2017, the global age-standardized prevalence rate of permanent teeth with untreated caries was 34.1%, affecting 2.5 billion people globally [2]. Therefore, caries control remains an enormous challenge for the global health service. 

Based on the extended ecological plaque hypothesis, caries are caused by the acidification of the dental biofilm ecosystem, which selectively increases the proportion of acidogenic and aciduric species in the microbiota composition, leading to an imbalance between the demineralization and remineralization processes that work toward net mineral loss [3]. This hypothesis inspired us to attempt to selectively eliminate conditional cariogenic microorganisms while sustaining salutary microorganisms within the flora in order to investigate whether these tactics could effectively regulate biofilms to further control caries. Among the complex microbial communities, *Streptococcus mutans* is well known as one of the predominant cariogenic agents in caries formation. The cariogenic potential of *S. mutans* mainly involves its ability to produce glucosyltransferases (Gtfs) that synthesize extracellular polysaccharides (EPS), its capacity to generate organic acids via carbohydrate metabolism (acidogenicity), and its potential to survive under low-pH conditions (aciduricity) [4,5]. 

Unlike *S. mutans*, *S. sanguinis* is a model commensal bacterium that is typically associated with the absence of dental caries and can interfere with *S. mutans* colonization by producing H_2_O_2_ via pyruvate oxidase SpxB [6]. Although both species coexist in the human oral biofilm, they have an antagonistic and competitive relationship, with a high level of one bacterium related to a low level of another [7]. It has been reported that the ratio of *S. mutans* to *S. sanguinis* is positively correlated with caries risk [8]. Therefore, a strategy of regulating biofilms with a relatively high level of *S. sanguinis,* or the suppression of opportunistic cariogenic bacteria, such as *S. mutans*, would be beneficial for the control of caries.

In recent years, despite most effective way to clean teeth manually using a brush, different types of antimicrobial agents have been used for dental plaque biofilm management to control caries. Chlorhexidine, with its broad-spectrum antimicrobial activity and plaque inhibitory potential, is well known as the gold standard oral antiseptic. However, it is associated with cytotoxicity, tastes bad, and has the possibility to reversible stain [9]. In addition, a simultaneous association with drug resistance and tolerance has been reported. For instance, reports suggest that chlorhexidine induces drug resistance in *S. mutans,* and the fungus *Candida albicans* may produce persisters after treatment with chlorhexidine [10,11]. In addition, the indiscriminate bactericidal effect of chlorhexidine would dramatically affect the microecology of oral microflora. 

Quaternary ammonium compounds, such as MDPB and DMAHDM, have also been studied in recent years. These nonreleasing antimicrobial agents exhibit long-term contact inhibition against bacteria. However, the high-concentration application of quaternary ammonium monomers results in significant cytotoxicity in mammalian cells [12]. It has desirable long-term antibacterial and anti-biofilm effects through a contact-killing mechanism when incorporated into dental composites [13,14]. However, quaternary ammonium salts can also induce persisters within *S. mutans* biofilms [15]. Therefore, despite the favorable results of these agents in terms of the antibiofilm effect described above, the side effects cannot be ignored. These drawbacks necessitate the need for a novel antimicrobial agent with a preponderance for selective inhibition and good biocompatibility without side effects, such as drug resistance and tolerance, to regulate biofilm formation. 

Irresistin-16 (IRS-16), a derivative of SCH-79797 with broad-spectrum antimicrobial properties, which possesses increased antimicrobial activity and reduced toxicity when compared to SCH-79797, is a potent and promising antibiotic candidate [16]. It is a dual-targeting compound with a pyrroloquinazolinediamine core with a biphenyl group targeting folate metabolism inhibition, and a biphenyl group on one side targeting membrane integrity disruption (Structures of IRS-16 was shown in Supplementary Materials Figure S1). Because of its unique dual-targeting mechanism of action, IRS-16 shows a remarkable capacity to suppress Gram-negative and Gram-positive pathogenic bacteria, albeit with undetectable antibiotic resistance using representative pathogens without *Streptococcus* spp. Meanwhile, it has been confirmed that IRS-16 displays enhanced efficacy and a robust capacity to eliminate infection in a mouse vaginal *Neisseria gonorrhea* (*N. gonorrhea*) model, with *N. gonorrhea* showing one of highest levels of drug resistance of all pathogens. However, to the best of our knowledge, no study has yet evaluated the effect of IRS-16 on biofilms. This study aimed to investigate the effects of this compound on dual-species biofilms with competitive relationships made up of *S. mutans* and *S. sanguinis* to further assess if it had similar function as arginine, which could regulate biofilm in an ecological way to manage dental caries potentially [17].

## 2. Results

### 2.1. IRS-16 Showed Disparate Efficiency on Two Types of Single-Species Biofilms

**Figure 1:**
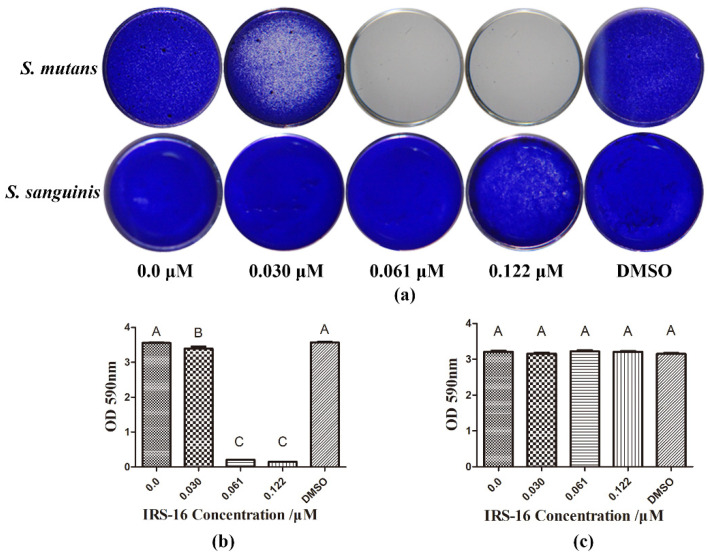
Antibiofilm formation activity of IRS-16 against single-species biofilm. (**a**) Crystal violet staining images of single-species biofilms under IRS-16. (**b**) Quantification of stained *S. mutans* biofilm. (**c**) Quantification of stained *S. sanguinis* biofilm. Values denoted by disparate letters indicate a significant difference (*p* < 0.05).

Results of crystal violet staining showed that IRS-16 at the concentrations of 0.061 and 0.122 μM exhibited marked anti-biofilm formation activity against *S. mutans*, whereas it had no remarkable anti-biofilm effect on *S. sanguinis*, even at high concentrations (Figure 1). These results indicated that IRS-16 exerted anti-biofilm formation activity in a species-dependent manner and had biofilm-regulating potential.

### 2.2. IRS-16 Modulated the Microbial Constitution of Dual-Species Biofilm

**Figure 2:**
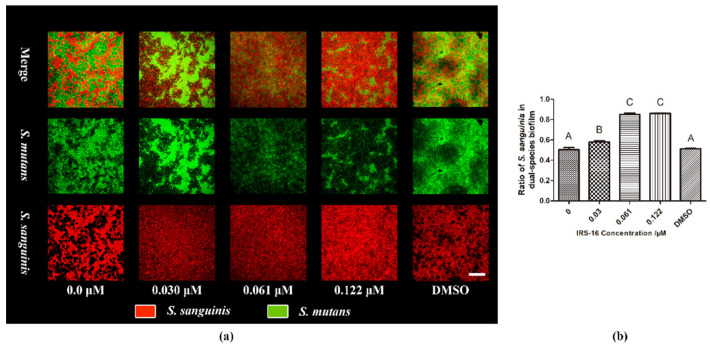
(**a**) Snapshots of dual-species biofilms by FISH (*S. mutans* is dyed green, *S. sanguinis* is stained red, scale bar is 50 μm). (**b**) Ratio of *S. sanguinis* within dual-species biofilms based on bacterial cover area. Data are presented as mean ± standard deviation; values denoted by different letters indicate a significant difference (*p* < 0.05).

FISH-labeled biofilms showed that the proportion of *S. sanguinis* increased within dual-species biofilms as the concentration of IRS-16 increased (Figure 2a). The coverage-based quantitative results show that the proportion of *S. mutans* was significantly decreased by IRS-16 compared to the control group. According to these results, IRS-16 caused the numbers of *S. mutans* to decrease, thereby impacting the proportion of *S. sanguinis*. As a result, IRS-16 changed the microbial composition of dual-species biofilms.

### 2.3. IRS-16 Decreased Metabolic Activity of Dual-Species Biofilms

**Figure 3:**
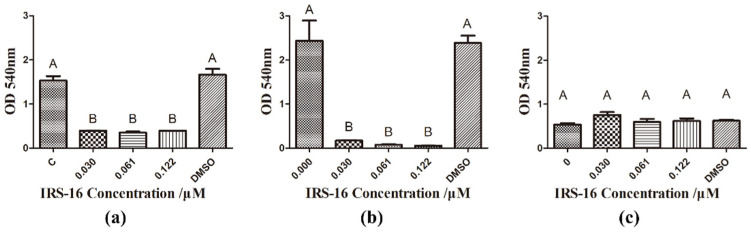
Metabolic activity of biofilms under IRS-16. (**a**) Metabolic activity of dual species biofilms revealed by the MTT method. (**b**) Metabolic activity of *S. mutans* biofilms. (**c**) Metabolic activity of *S. sanguinis* biofilms. Values denoted by different letters indicate a significant difference (*p* < 0.05).

The effects of IRS-16 on the metabolic activity of biofilms using the MTT assay are shown in Figure 3. In dual-species biofilms, IRS-16 concentrations of 0.03 μM, 0.061 μM and 0.122 μM all demonstrated significantly reduced OD values. Similarly, when treated with different concentrations of IRS-16, *S. mutans* biofilms exhibited a noteworthy reduction in OD values with increasing compound concentrations. However, *S. sanguinis* biofilms showed no remarkable changes.

### 2.4. IRS-16 Inhibited Lactic Acid Generation of Dual-Species Biofilms

**Figure 4:**
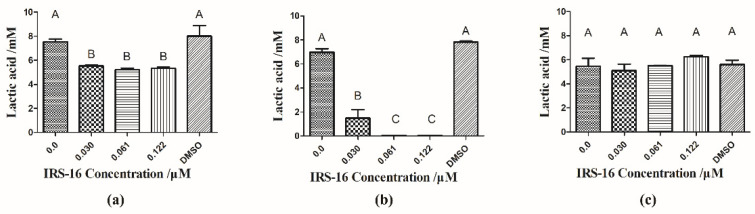
Lactic acid generation in biofilms affected by IRS-16. (**a**) Lactic acid generation of dual-species biofilms. (**b**) Lactic acid generation in *S. mutans* biofilms. (**c**) Lactic acid generation in *S. sanguinis* biofilms. Values denoted by disparate letters indicate a significant difference (*p* < 0.05).

The lactic acid generation within the biofilms is shown in Figure 4. In dual-species biofilms, the IRS-16 treatment groups produced significantly less lactic acid than the control group (*p* < 0.05). Likewise, in the *S. mutans* biofilm, 0.03 μM IRS-16 had already exhibited marked suppressive effects on lactic acid production. Moreover, at IRS-16 concentrations of 0.061 μM and 0.122 μM, almost no lactic acid was produced. On the other hand, no significant difference was found in lactic acid production after IRS-16 treatment in *S. sanguinis* biofilms. Thus, lactic acid production was significantly inhibited by IRS-16 in dual-species biofilms.

### 2.5. IRS-16 Reduced EPS Synthesis in Dual-Species Biofilms

**Figure 5:**
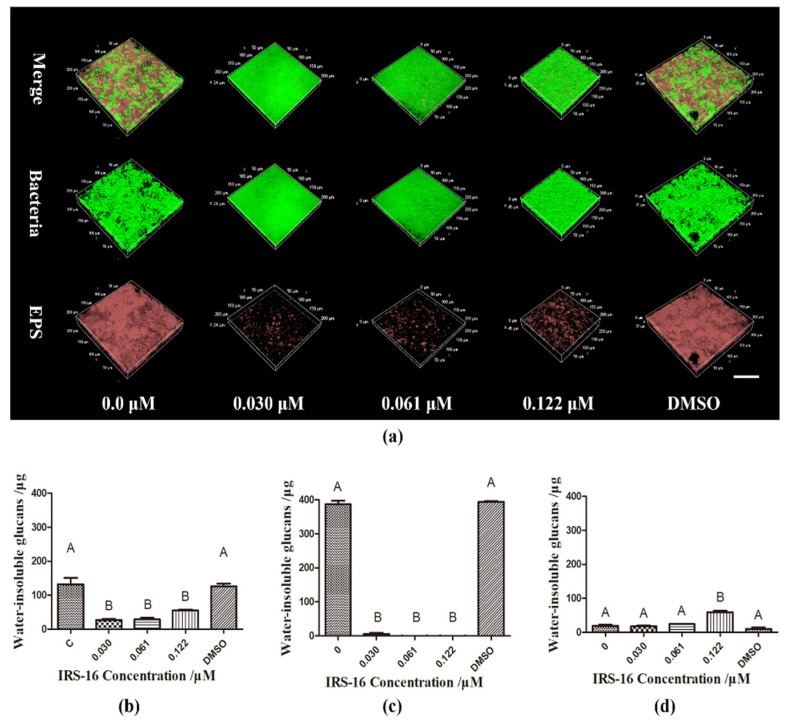
Bacteria and EPS distribution within the dual-species biofilms. (**a**) Bacteria and EPS dye of dual-species biofilms under the effect of IRS-16 (Bacteria are marked green and EPS is marked red, scale bar is 50 μm). (**b**) Water-insoluble glucans in dual-species biofilms, (**c**) *S. mutans* biofilms and (**d**) *S. sanguinis* biofilms. Values denoted by disparate letters indicate a significant difference (*p* < 0.05).

Figure 5a shows bacterial and EPS distribution within the dual-species biofilms. Bacteria and EPS were dyed green and red, respectively. In the control and DMSO groups, more microorganisms and EPS were observed. In the IRS-16-treated groups, bacteria and EPS decreased significantly. The results of the quantitative analysis of water-insoluble glucans synthesis in dual-species biofilms are shown in Figure 5b. Compared to the control and DMSO groups, water-insoluble glucans production was significantly reduced in 0.03, 0.061, 0.122 μM IRS-16-treated groups. Moreover, abundant water-insoluble glucans production was observed in the control and DMSO groups in the *S. mutans* biofilm. This was almost absent in the IRS-16-treated group (Figure 5c). Conversely, regardless of treatment, only a small amount of water-insoluble glucans was detected in *S. sanguinis* biofilms, and this was significantly elevated in the 0.122 μM IRS-16-treated group (Figure 5d). These results imply that IRS-16 could reduce EPS generation by bacteria in dual-species biofilms.

### 2.6. IRS-16 Influenced the Bacterial Growth of the Two Species

**Figure 6:**
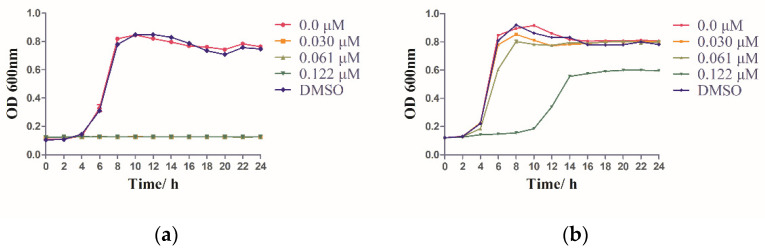
Growth curves; 24 h growth curves of (**a**) *S. mutans* and (**b**) *S. sanguinis*.

When exposed to various concentrations of IRS-16 (0.030, 0.061 and 0.122 μM), the bacterial growth of *S. mutans* was inhibited significantly when compared to that of blank control and solvent control (Figure 6a). In contrast, IRS-16 concentrations of 0.030 and 0.061 μM hardly affected the proliferation of *S. sanguinis*, but the 0.122 μM IRS-16 concentration displayed a partial inhibitory effect (Figure 6b). These results indicate that IRS-16 had a greater impact on the growth of *S. mutans* than *S. sanguinis*.

### 2.7. IRS-16 Exhibited No Remarkable Toxicity to L929 Cells

**Figure 7:**
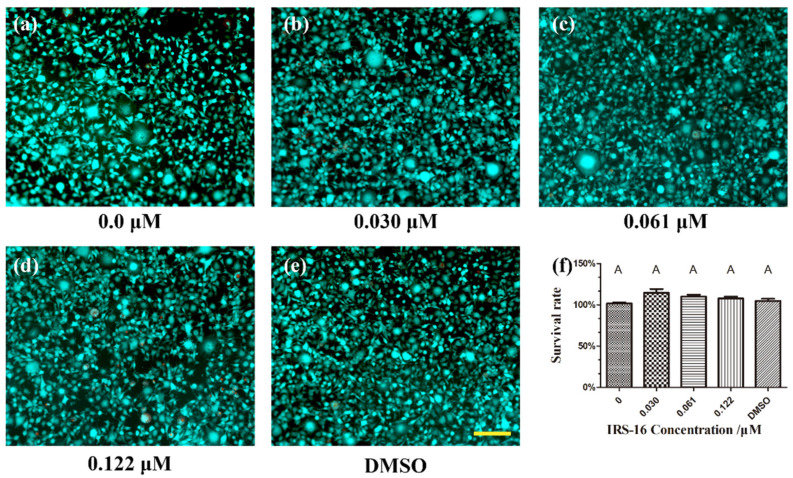
Cytotoxicity evaluation of IRS-16. (**a**–**e**) Live/dead cell assay of L929 cells under different treatments. Live cells are green, dead cells are red and scale bar is 100 μm. (**f**) Cell viability based on CCK-8 assay. Values denoted by disparate letters indicate a significant difference (*p* < 0.05).

The cytotoxicity of IRS-16 was assessed using mouse fibroblast L929 cells under different treatments. As shown in Figure 7a–e, in the live/dead cell assay, viable cells (green) were detected in abundance in all the concentration groups, and dead cells (red) were scarce. The CCK-8 assay (Figure 7f) indicated that IRS-16 did not induce significant cytotoxicity in L929 cells among the experimental and control groups. The results demonstrate that IRS-16 has low cytotoxicity, and potential for use in clinical biofilm control applications.

## 3. Discussion

Dental caries is a biofilm-mediated chronic disease. Therefore, efficient biofilm management is key to dental caries control. Antimicrobial agents have been used as an indispensable supplement for the control of dental plaques for many years [18]. However, with the discovery of drawbacks such as drug tolerance and resistance in recent years, the application of antimicrobial agents has been somewhat limited. Furthermore, as reported by Marsh et al., control without killing based on the ecological plaque hypothesis would be a good approach to control caries through biofilm regulation [19]. 

Our in vitro investigations explored for the first time the effects of IRS-16 on dual-species biofilms developed by the opportunistic pathogen *S. mutans* and commensal microbe *S. sanguinis*. We found that IRS-16 regulated the biofilm by not only changing its composition, but also by suppressing cariogenic virulence, including acid and EPS synthesis. The biofilm regulatory effect of IRS-16 was similar to that of arginine which could also regulate biofilm in an ecological way [17]. Both the minimum inhibitory concentration (MIC) and the minimum biofilm inhibitory concentration (MBIC) of IRS-16 against *S. sanguinis* (1.953 μM for MIC, 1.953 μM for MBIC) were much higher than those of *S. mutans* (0.122 μM for MIC, 0.061 μM for MBIC). Furthermore, for *S. mutans* biofilms, IRS-16 significantly inhibited biofilm formation, as well as metabolic activity and virulence, including lactic acid and EPS generation. In contrast, IRS-16 had little impact on the growth and cariogenic virulence of *S. sanguinis* biofilms at the corresponding concentrations. The dissimilar sensitivities of *S. mutans* and *S. sanguinis* to IRS-16 were partly due to the different levels of bacterial growth inhibition. This selective antibacterial effect implied that the application of IRS-16 might have the potential of selectively suppressing cariogenic pathogens such as *S. mutans* and sustaining a relative intact micro-ecosystem instead of eliminating microflora simultaneously [18]. From an ecological caries-prevention perspective, the competitive advantage of commensal *S. sanguinis* over cariogenic *S. mutans* relates to the transition of the microbial equilibrium in biofilms from a high cariogenic risk to a healthy state, and enables a reduction in the synthesis of cariogenic virulence factors that contribute to the dysbiosis of dental plaque biofilm in situ [20,21].

EPS, which can be synthesized by streptococcal Gtfs by utilizing sucrose, is recognized as a critical cariogenic factor because it can help bacteria adhere to the tooth, create a distinct acidic architecture for microbes to persist, provide protection from antimicrobials, and aid in nutrient acquisition [22]. Therefore, the precise inhibition of EPS biosynthesis has been recommended as an effective and specific strategy to disrupt biofilm formation, and thus suppress dental caries development [22,23]. Our results revealed that IRS-16 could significantly reduce EPS, especially water-insoluble glucan production in single *S. mutans* and dual-species biofilms. According to previous studies, in the initial colonizing community, *S. mutans* was the main producer of EPSs in the presence of sucrose among other organisms [5,23,24]. Therefore, the altered compositions of *S. sanguinis* and *S. mutans,* and the inhibition of the EPS synthesis of *S. mutans* alone caused by IRS-16, were probably responsible for the decrease in total EPS synthesis in dual-species biofilms. Interestingly, a significant increase in water-insoluble glucan synthesis was detected in *S. sanguinis* biofilms when they were treated with a concentration of 0.122 μM of IRS-16. This might be due to the fact that *S. sanguinis* produced more exopolysaccharide matrix to encase the biofilm cells and increase the diffusive resistance, thus helping it resist the stress induced by IRS-16 [25]. Moreover, total EPS reduction might also be associated with the reduction in the metabolic activity of the *S. mutans* biofilm [26].

It is well known that bacterial acidogenicity is a primary virulence factor related to cariogenicity [26]. A low pH could swing the balance of the dissolution reaction for hydroxyapatite to demineralization and cause the subsequent formation of caries [27]. When subjected to excessive amounts of sugar, *S. mutans* produces lactic acid by metabolizing fermentable carbohydrates through glycolysis [28]. In the present study, IRS-16 had the capacity to depress lactic acid generation in *S. mutans* and dual-species biofilms but had no significant effect on *S. sanguinis* biofilms. Although *S. sanguinis* can also produce lactic acid by metabolizing glucose, *S. mutans* has a higher adenosine triphosphate-glucose phosphotransferase activity, with a stronger acid generation ability than *S. sanguinis* [29]. Therefore, the stronger inhibitory effect of IRS-16 on *S. mutans* resulted in a reduction in lactic acid production in biofilms containing *S. mutans*. In addition, lactic acid generation may indicate biofilm metabolic activity to some extent [30,31]. The similar trends between these two parameters, along with their well-established biocompatibility, further support the biomedical application potential of IRS-16 to inhibit the metabolic activity of cariogenic biofilms.

The specific mechanisms underlying the selective antibacterial effects of IRS-16 on dual-species biofilms in this study remain largely unknown. According to Martin et al. [16], the antimicrobial mechanisms behind IRS-16 are enhanced membrane targeting activity and increased dihydrofolate reductase inhibition. These unique dual-targeting mechanisms of action endow IRS-16 with potent antibacterial properties and inhibited Gram-negative (such as *N. gonorrhea*, *Escherichia coli*) and Gram-positive (*Bacillus subtilis*, *Staphylococcus aureus*, *Enterococcus faecalis*) pathogens [16]. A separate study screened three new analogs based on trimetrexate, a human dihydrofolate reductase inhibitor, and indicated that the compounds could selectively inhibit the *S. mutans* biofilm while failing to suppress *S. sanguinis* and *S. gordonii* biofilm formation [32]. The findings were consistent with the results of this study to some extent, suggesting that IRS-16 might also selectively inhibit *S. mutans* partly resulting from targeting dihydrofolate reductase [32]. GH12, a newly designed antimicrobial peptide, whose possible antimicrobial mechanism was influencing the cell membrane, showed a stronger antibacterial effect on *S. mutans* than *S. sanguinis* [33]. The dissimilar susceptibility of these two species against IRS-16 in this study might be partly similar to their different sensitivity to GH12, which probably due to their differences in cell membrane composition [33]. Another possible ecological reason for the changes in the composition of the dual-species biofilm might be that IRS-16 helped *S. sanguinis* to inhibit competing species, thereby allowing *S. sanguinis* to gain the advantage of initial colonization over *S. mutans*. However, the specific molecular mechanisms of IRS-16 in the selective inhibition of *S. mutans* biofilm remain to be investigated. Furthermore, whether oral bacteria including *S. mutans* and *S. sanguinis* would induce drug resistance is needed to be further studied. Although IRS-16 showed a regulatory effect on dual-species biofilms consisting of *S. mutans* and *S. sanguinis*, the defined biofilm model did not effectively represent extremely complicated dental plaque. A comprehensive study estimating the ecological effects of IRS-16, such as arginine, is needed [34].

## 4. Materials and Methods

### 4.1. Microbial Strains and Culture Conditions

*S. mutans* UA159 and *S. sanguinis* ATCC 10556 were incubated overnight in Brain Heart Infusion Broth (BHI, Oxoid, Basingstoke, UK) for bacterial proliferation (37 °C, 5% CO_2_). Commercially acquired IRS-16 was dissolved in dimethyl sulfoxide (DMSO) at a concentration of 25 mM as a stock solution. For construction of the growth curve, we used BHI as the culture media. For biofilm formation, BHI medium together with 1% sucrose (*m*/*v*) was used.

For the crystal violet assay, 10^6^ CFU/mL of overnight bacterial culture was added to a 96-well plate together with gradient IRS-16 for 24 h (200 μL culture volume). For other biofilm formation associated assays, sterile glass slides were placed in the 24-well plate, and bacterial solutions of *S. mutans*, *S. sanguinis* or their mixture were inoculated at 10^6^ CFU/mL (2 mL culture volume) together with different concentrations of IRS-16 for 24 h. A group cultured without IRS-16 (0.0 mM) was used as the blank control, and 1% DMSO was used as the solvent control. IRS-16 was synthesized from PharmaAdvance, China.

### 4.2. Crystal Violet Test

A crystal violet test was performed to investigate the biofilm biomass [35]. Briefly, single-species biofilms formed in 96-well plates were settled in methanol (15 min), followed by air-drying at room temperature. After biofilms were stained with 100 μL 0.1% (*w*/*v*) crystal violet solution (20 min), nonspecific staining was cleaned with poly butylene succinate (PBS). Biofilms were then observed under a stereomicroscope (Nikon SMZ800, Nikon Corporation, Tokyo, Japan). For quantification analysis, 33% acetic acid (200 μL) was applied to dissolve the dyed crystal violet, and the absorbance at 590 nm was determined (SpectraMax M5, Molecular Devices, San Jose, CA, USA).

### 4.3. Fluorescence In Situ Hybridization (FISH)

FISH was conducted to monitor biofilm composition [26]. In brief, dual-species biofilms were fixed with 4% paraformaldehyde for 12 h, rinsed twice with distilled water, and dried at 46 °C. Then, the samples were treated with lysozyme solution (50 mM EDTA, 100 mM Tris−HCl, 30 mg/mL lysozyme, pH 8.0) for 30 min at 37 °C. After being dehydrated in gradient ethanol (50%, 80%, and 96%) for 3 min each and dried at 46 °C for 10 min, they were treated with hybridization buffer containing species-specific probes (Invitrogen Investment, Shanghai, China) and incubated for 90 min at 46 °C away from light (Nucleotide Sequences of species-specific probes are listed in Table 1). A confocal laser scanning microscope (CLSM, Nikon A1, Nikon Corporation, Japan) with a 60× oil immersion lens was employed to observe the stained biofilms. Five random fields of each biofilm were used for the semiquantitative analysis. Biofilm quantification was performed according to the coverage area using Image-Pro Plus software (version 6.0, Media Cybernetics, Inc., Silver Spring, MD, USA).

### 4.4. MTT Assay

A 2, 5-diphenyltetrazolium bromide (MTT) assay was used to detect the vitality of biofilms, as previously described [37]. After washing with PBS twice, the 24 h biofilms were placed into another 24-well plate. Aliquots of 1 mL of 0.5 mg/mL MTT were then added to cover the biofilm. After 1 h of incubation in 5% CO_2_ at 37 °C, the biofilms were transferred to another 24-well plate. Next, 1 mL of dimethyl sulfoxide (DMSO) was incubated with the MTT-stained biofilm for 20 min. Finally, the OD of aliquots of 200 μL solubilized MTT solution at 540 nm were read using a microplate reader spectrophotometer in a 96-well plate (SpectraMax M5, Molecular Devices, USA).

### 4.5. Lactic Acid Detection

Lactic acid measurements were conducted to monitor lactic acid generation [38]. After 24 h of biofilm formation, the biofilm slides were rinsed with cysteine peptone water and then placed into a new 24-well plate. Aliquots of 1.5 mL buffered peptone water (BPW) containing 0.2% sucrose were used to produce acid for 3 h in 5% CO_2_ at 37 °C. The lactate concentration in the BPW solution was detected via the lactate dehydrogenase reaction, for which, 190 μL reaction buffer (0.45 M glycine, 0.36 M hydrazine sulfate, and 2.73 mM NAD^+^) was mixed with 10 μL BPW solution containing lactate in 96-well plates, and the absorbance was read at 340 nm. Then, 10 µL LDH solution (1 mg/mL) was added, and incubation was performed at room temperature for 1 h, followed by absorbance reading at 340 nm. The difference in the OD values between before and after 1 h of incubation was used to compute the final lactic acid production with standard curves.

### 4.6. Bacterial/Extracellular Polysaccharide (EPS) Dye

Bacterial/EPS dye was prepared as previously described [39]. Briefly, bacteria were cultured with Alexa Fluor 647-dextran conjugate (2.5 μM, Invitrogen Molecular Probes, Carlsbad, CA, USA) for 24 h in EPS dye. Then, bacterial cells within biofilms were stained with SYTO-9 (2.5 μM, Invitrogen Molecular Probes, Carlsbad, CA, USA) for 30 min, and then scanned with a layer thickness of 1 μm, and a three-dimensional (3D) image was reestablished using confocal laser scanning microscopy (Nikon A1, Nikon Corporation, Japan). Polysaccharides and bacteria were stained red and green independently within the 3D biofilms.

### 4.7. Water-Insoluble Exopolysaccharide Determination

The anthrone method was applied for the quantitative analysis of water-insoluble EPS generation in 24 h biofilms, as described previously [40]. In brief, after the collection of biofilms by centrifugation (4000 rpm, 10 min), sediments were resuspended in 0.4 M NaOH (1 mL) followed by another centrifugation (4000 rpm, 10 min). The supernatant (300 μL) was reacted with 900 μL anthrone reagent for 6 min at 95 °C in a water bath. The absorbance at 625 nm was detected using a microplate reader (SpectraMax M5, Molecular Devices, USA). Water-insoluble exopolysaccharides were displayed based on the standard curves.

### 4.8. Growth Curve

The growth curve was used to investigate the effect of IRS-16 on the growth of *S. mutans* and *S. sanguinis* [41]. Briefly, bacteria were overnight cultured. Then, they were added in a 96-well microtiter plate and exposed to IRS-16, yielding a final concentration of 10^6^ cell-forming cells (CFU)/mL at a final volume of 200 μL. After that, they were incubated for 24 h. The OD was spectrophotometrically monitored at 600 nm every 2 h for up to 24 h, using a microplate reader (SpectraMax M5, Molecular Devices, USA).

### 4.9. Live/Dead Cellular Viability Assay

The cytotoxicity of IRS-16 was determined using a live/dead viability assay [23]. Briefly, 1 × 10^5^ cells/well mouse fibroblast L929 cells were cultured overnight in a 96-well plate in Dulbecco’s modified eagle’s medium (DMEM, Gibco, Eggenstein, Germany) together with 10% fetal bovine serum (Gibco, Eggenstein, Germany). After the cells attached to the 96-well plate, they were exposed to IRS-16 and DMSO solution for 24 h. Then, each well was treated with the Calcein-AM/PI double staining kit (Dojindo, Tokyo, Japan) according to the manufacturer’s instructions. The stained L929 cells were assessed using fluorescence microscopy (Axio Vert.A1, ZEISS, Jena, Freistaat Thüringen, Germany), in which vital cells and dead cells appeared green and red, respectively.

### 4.10. CCK-8 Assay

A Cell Counting Kit-8 (CCK-8, Dojindo, Kumamoto, Japan) was used to quantify the viability of cells treated with IRS-16 [42]. Briefly, mouse fibroblast L929 cells were seeded in the same manner as the live/dead cellular viability assay. After treatment with different concentrations of IRS-16 and DMSO solution for 24 h, the CCK-8 was incubated with the cell culture for 1 h. The absorbance was determined at 450 nm (SpectraMax M5, Molecular Devices, USA), and the survival rate was calculated based on the 0 μM IRS-16 group.

### 4.11. Statistical Analysis

All tests were conducted at least thrice. Statistical analysis was performed using one-way analysis of variance (ANOVA), followed by Tukey’s multiple-comparison test. Values denoted by disparate letters indicated a significant difference (*p* < 0.05). Statistical analyses were performed using the SPSS software 16.0 (IBM Corp., Armonk, NY, USA).

## 5. Conclusions

IRS-16 has selective inhibition and latent regulatory effect on dual-species biofilms consisting of *S. mutans* and *S. sanguinis*, including microbial composition modification, and the inhibition of critical cariogenic virulence factors. In addition, it exhibited low cytotoxicity against mouse fibroblast L929 cells. Based on the findings of this study, IRS-16 has great potential for ecological applications in the control of dental caries.

## Figures and Tables

**Table 1:** Nucleotide Sequences of species-specific probes.

Probes	Nucleotide Sequence (5′–3′)	Reference
*S. mutans* *S. sanguinis*	Alexa Fluor 488-5′-ACTCCAGACTTTCCTGAC-3′ Alex Fluor 594-5′-GCATACTATGGTTAAGCCAC AGCC-3′	[36]

## Data Availability

All data used to support this study are available from the corresponding author on reasonable request.

## Supplementary Materials

The following supporting information can be downloaded at: https://www.mdpi.com/article/10.3390/pathogens11010070/s1, Figure S1: Structures of IRS-16.
