# Supplementary figures and images for: Regulatory Effect of Irresistin-16 on Competitive Dual-Species Biofilms Composed of Streptococcus mutans and Streptococcus sanguinis
Source: Pathogens. 2022 Jan 6;11(1):70. doi: 10.3390/pathogens11010070 (PMC8779588; doi:10.3390/pathogens11010070)

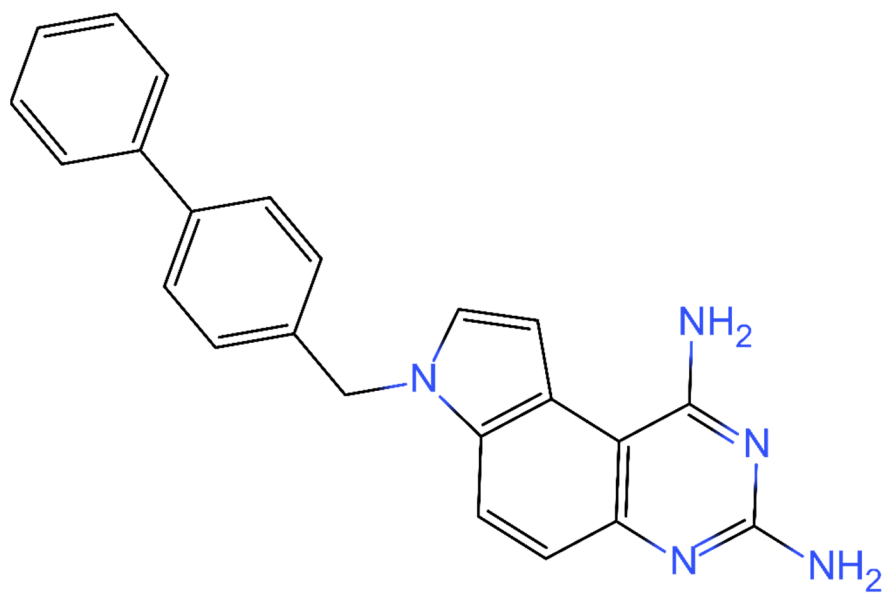

**Supplementary Material Figure S1.** Structures of IRS-16.

Supplement: Supplementary file 1 [file pathogens-11-00070-s001.zip › pathogens-1506828-supplementary.pdf]
